# Fruit Peels: Food Waste as a Valuable Source of Bioactive Natural Products for Drug Discovery †

**DOI:** 10.3390/cimb44050134

**Published:** 2022-04-30

**Authors:** Hidayat Hussain, Nilufar Z. Mamadalieva, Amjad Hussain, Uzma Hassan, Aisha Rabnawaz, Ishtiaq Ahmed, Ivan R. Green

**Affiliations:** 1 Department of Bioorganic Chemistry, Leibniz Institute of Plant Biochemistry, Weinberg 3, D-06120 Halle (Saale), Germany; 2 Institute of the Chemistry of Plant Substances of the Academy Sciences of Uzbekistan, Tashkent 100170, Uzbekistan; nmamadalieva@yahoo.com; 3 Department of Chemistry, University of Okara, Okara 56130, Pakistan; aishanawaz2937@gmail.com; 4 Institute of Chemical Sciences, University of Peshawar, Peshawar 25120, Pakistan; umigul5@gmail.com; 5 Department of Chemical Engineering and Biotechnology, University of Cambridge, Cambridge CB3 0AS, UK; ishtiaq.chem@googlemail.com; 6 Department of Chemistry and Polymer Science, University of Stellenbosch, Private Bag X1, Matieland, Stellenbosch 7600, South Africa; irg@sun.ac.za

**Keywords:** fruit peels, natural product, antimicrobial, antioxidant, food industry

## Abstract

Fruits along with vegetables are crucial for a balanced diet. These not only have delicious flavors but are also reported to decrease the risk of contracting various chronic diseases. Fruit by-products are produced in huge quantity during industrial processing and constitute a serious issue because they may pose a harmful risk to the environment. The proposal of employing fruit by-products, particularly fruit peels, has gradually attained popularity because scientists found that in many instances peels displayed better biological and pharmacological applications than other sections of the fruit. The aim of this review is to highlight the importance of fruit peel extracts and natural products obtained in food industries along with their other potential biological applications.

## 1. Introduction

Approximately 89 million tons of food waste is produced in the European Union, and this figure is anticipated to increase by a factor of 40 in the future [1]. Fruits and vegetables are considered to be basically used food products being either fully cooked, nominally cooked or uncooked [1]. It has been found that the processing of vegetables and fruits alone produces a notable waste of 25–30% of the total product. Furthermore, peels, pomace, rind and seeds are considered to be among the most common wastes. In spite of this, material contains valuable biologically active molecules including enzymes, carotenoids, oils, polyphenols and vitamins. In point of fact, these bioactive molecules demonstrated their significant industrial application including as a food to generate edible films along with probiotics and other industrial applications to develop value-added products [1].

It has been reported that large amounts of secondary metabolites are present in fruit and vegetable wastes and these waste materials have been studied for phenolic molecules, dietary fibers and other biologically active metabolites by extraction [1,2]. Scientific investigations revealed that phytochemicals and essential nutrients are largely present in the peels, seeds, fruits and vegetables [1]. For example, the skin of grapes, avocados, lemons along with seeds of mangoes and jackfruits comprise up to a 15% larger phenolic content than fruit pulp [3,4]. The fruit and vegetable wastes could thus be employed to obtain biologically active metabolites that could be utilized in food industries, cosmetics, food, pharmaceutical and textile industries. The proper utilization of fruit peels will not only resolve the large number of environmental problems, but this strategy will improve health through enriched food products comprising health-enhancing molecules. To the best of our knowledge no comprehensive review has been published about natural products which were isolated from fruit peels and our review mainly focus on the natural products which are purely isolated and their biological effects but not detected via mass techniques. Indeed, some minireviews have been published about the fruit peel crude extracts and biological activities or invidual reviews of fruit peels and their natural products [1,5,6,7,8,9,10,11,12,13,14,15,16,17,18,19]. 

## 2. Traditional Uses of Fruit Peels

Citrus (C) peels are not fully utilized and these waste materials contain highly valuable bioactive molecules [20]. In addition, fruits and their peels are folklorically used to treat cough, digestive problems, infection, muscle pain and skin inflammation [21,22]. These folkloric preparations are formulated from the peel, fruit and flowers [23]. Notably, peels of *C. reticulata* and *C. unshiu* are used under the trade name “Chimpi” in Japan as crude drugs. Similarly, *C. aurantium* dried peels are employed as the popular folkloric drug “Touhi.” [20,24]. The “tangerine”, a citrus fruit, is considered as one of the most popular foods in many countries around the world. Citrus peel, named “Chenpi” in China, has been employed as a medicine to treat gastrointestinal and respiratory diseases. In addition, the peel of a tangerine is used in drinks and baking in Western countries as an aromatic spice [25,26,27]. Citrus fruit peels are reported to be used in Chinese medicine to treat muscle pain, stomachache, cough, skin inflammation and high blood pressure [28]. Wampee (*Clausena lansium*) peels are used to treat bronchitis and stomachache in China and Indian folk medicine [29]. The fruit peels of wampee (*Clausena lansium*) are reported to be utilized for stomachic, bronchitis and as a vermifuge [29] while fruit peels of Jaboticaba (*Plinia peruviana*) are employed for diarrhea, skin irritation, hemoptysis and asthma [30].

## 3. Coumarins

**Figure 1:**
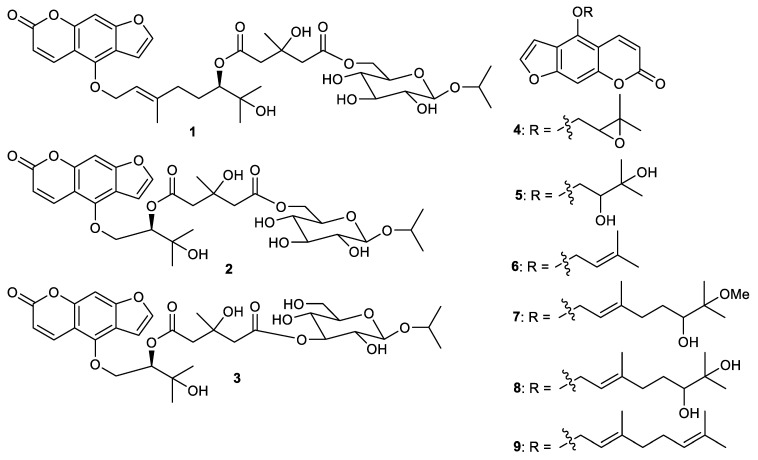
Structures of coumarins **1**–**9**.

Furanocoumarins citrusosides B-D (**1**–**3**) (Figure 1), oxypeucedanin (**4**), oxypeucedanin hydrate (**5**), 6′-hydroxy-7′-methoxybergamottin (**6**), isoimparatorin (**7**), bergomottin (**8**), 6′,7′-dihydroxybergamottin and (**9**) were isolated from the fruit peels of *Citrus hystrix* [31]. Furanocoumarins **7** and **8** exerted significant butyrylcholinesterase inhibition with IC_50_: 11.2 and 15.4 µM, respectively. On the other hand, furanocoumarins **4** and **6** were also active, but to a lesser extent towards butyrylcholinesterase enzyme with inhibitions of IC_50_: 63.0 and 23.1 µM, respectively. In addition, coumarin **1** possesses a weak inhibition with IC_50_: 339 µM while compounds **3**, **5** and **9** were not active [31]. Coumarins **5** and **8** are also reported from the fruit peels of *Citrus hystrix* (Kaffir lime) and it was demonstrated that **5** exhibited iNOS production in RAW264.7 cells, lipopolysaccharide-interferon gamma-induced NO and COX-2 production in HCT116 and HT-29 cells with IC_50_: 22.5, 18.2, 23.2 and 22.2 μg/mL, respectively, whereas compound **8** had IC_50_ values of 18.6, 16.1, 17.5 and 18.1 μg/mL, respectively [32].

**Figure 2:**
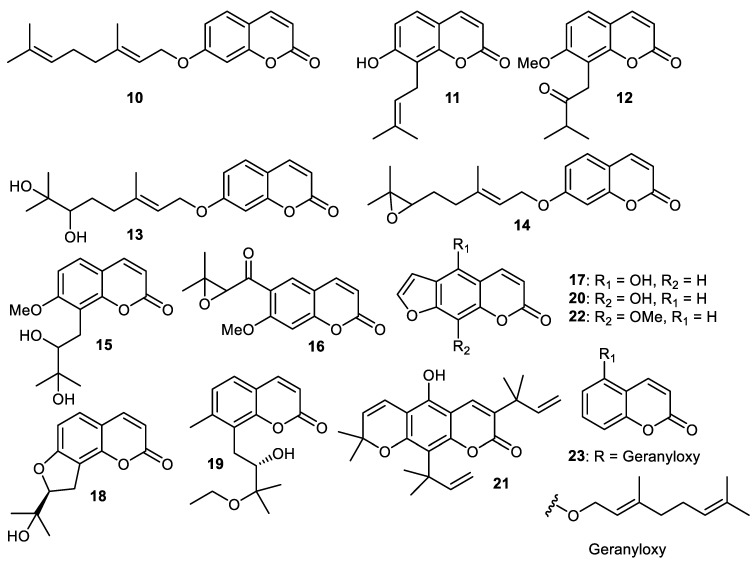
Structures of coumarins **10**–**23**.

A literature survey illustrated that the peels of *C. grandis* fruits are employed in traditional Chinese medicine to treat exhaustion, cancer and the common cold [33]. Coumarin **8**, auraptene (**10**) (Figure 2), osthenol (**11**), isomeranzin (**12**), marmin (**13**), epoxyaurapten (**14**), meranzin hydrate (**15**), 7-hydroxy-8-(2′-hydroxy-3′-methylbut-3′-enyl) coumarin (**16**), bergaptol (**17**), columbianetin (**18**) and yuehgesin-C (**19**) are all produced by *C. grandis* peels. It is interesting to note that coumarins **8**, **12** and **14** inhibited the generation of the superoxide anion and elastase activity with IC_50_: 6.02, 3.89 and 7.57 µM, respectively [34].

Wampee fruit (*Clausena lansium*) produces 8-hydroxypsoralen (**20**) which exerted good antioxidant effects. It also exhibited potent cytotoxic effects towards human liver cancer (HepG2: IC_50_: 0.34 µg/mL), lung cancer (A549: IC_50_: 28.2 µg/mL) and cervical cancer (HELA: IC_50_: 0.013 µg/mL) [29]. Coumarin **21** was isolated from *C. reticulata* fruit peels [35] while coumarins **22** and **23** were produced by peels of *C. reticulate* [36]. 

**Figure 3:**
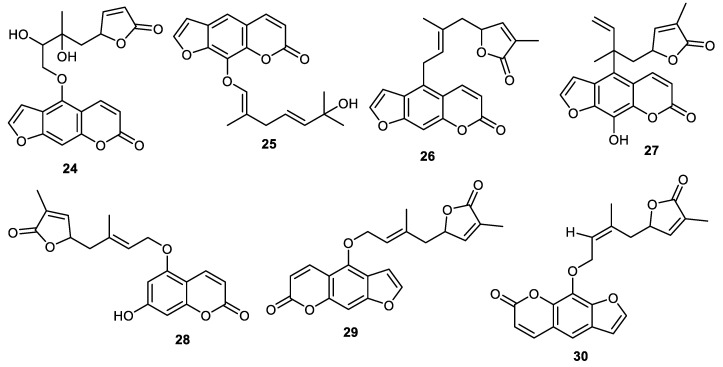
Structures of coumarins **24**–**30**.

Dihydromyric (**24**) and 2″,3″-dihydroxyanisolactone (**25**) (Figure 3) were reported from the peels of *Clausena lansium* and coumarin **24** illustrated α-glucosidase effects while compound **25** illustrated antibacterial effects towards *Staphylococcus aureus* [37]. The peels of *Clausena lansium* produced clauslactone V (**26**), clauslactoneW (**27**), clausenalansimin B (**28**), anisolactone (**29**) and wampetin **(30**) and all compounds demonstrated better α-glucosidase effects (IC_50_: 0.12 to 0.72 mM) than standard acarbose (IC_50_: 1.55 mM) [38].

## 4. Quinone and Phenolic Glycosides

**Figure 4:**
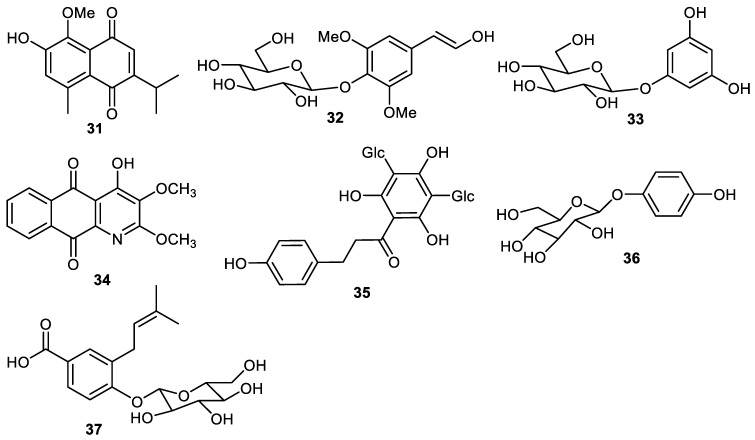
Structures of quinone and phenolic glycosides **31**–**38**.

Quinone **31** (Figure 4) is produced by the fruit peel of *Elaeagnus rhamnoides* [39] and later this same compound was also reported from *Rumex aquaticus* [40]. It was found that compound **31** displayed antiviral effects at a 50 µM concentration [39]. Eleutheroside B (**32**) and phlorin (**33**) are produced by the peels of *C. grandis* [34] and scorazanone (**34**) was reported from peels of *Goniothalamus scortechinii* [41]. 3′,5′-Di-*C*-β-glucopyranosylphloretin (**35**) was produced by the peels of *Fortunella japonica* [42] while arbutin (**36**) and malaxinic acid (**37**) were reported from *Pyrus pyrifolia* (Asian pear) [43]. 

## 5. Sesquiterpenes

**Figure 5:**
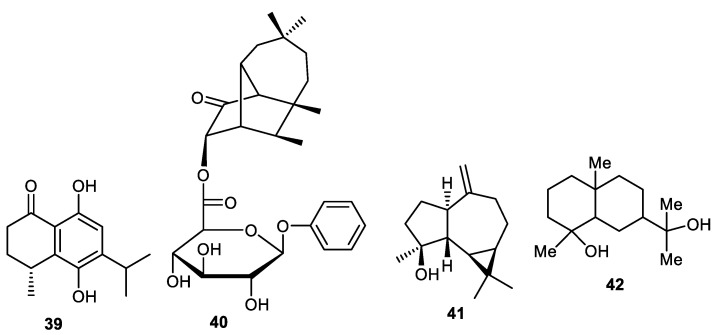
Structures of sesquiterpenes **39**–**42**.

*Elaeagnus rhamnoides* was reported to contain *nor*-sesquiterpene **39** (Figure 5) from its fruit peel and its absolute configuration was measured via the TDDFT-ECD method. In antiviral screening, compound **39** was found to cause a 2 log_10_ reduction in HSV-2 yield and additionally possesses antiviral effects as determined in the qPCR method [39]. A new sesquiterpene glycoside **40** was found to be produced by the fruit peels of *C. limon* [44]. Spathulenol (**41**) was isolated from the peels of *Annona squamosal* [45] while crytomeriodiol (**42**) was produced by peels of *Goniothalamus scortechinii* [41]. 

## 6. Naphthalene and Betacyanins 

**Figure 6:**
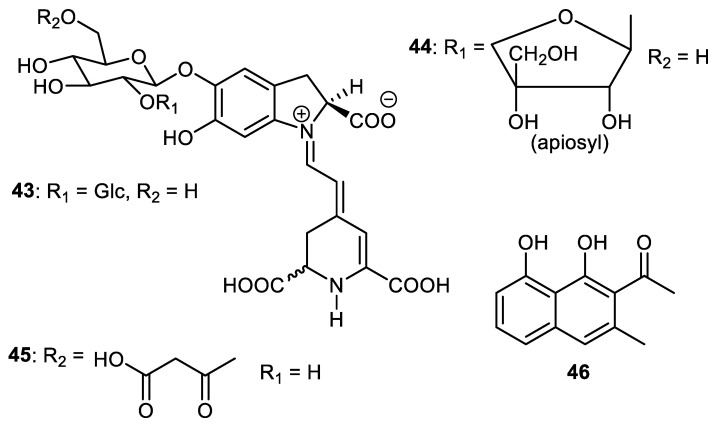
Structures of naphthalene and betacyanins **43**–**46**.

Betacyanins, 2′-O-glucosylbetanin (**43**), 2′-O-apiosylbetanin (**44**) and 2′-O-apiosylphyllocactin (**45**) (Figure 6) are produced by the fruit peels of *Hylocereus* sp. [46]. Musizin (**46**), a naphthalene derivative, was isolated from the peel of *Elaeagnus rhamnoides* and this compound exerted antiviral effects and caused a 2.33 log_10_ reduction at a 12.5 µM concentration [39]. Interestingly enough, the anti-HSV-2 effect of musizin (**46**) was the same as that reported by Gescher et al. [47].

## 7. Alkaloids

**Figure 7:**
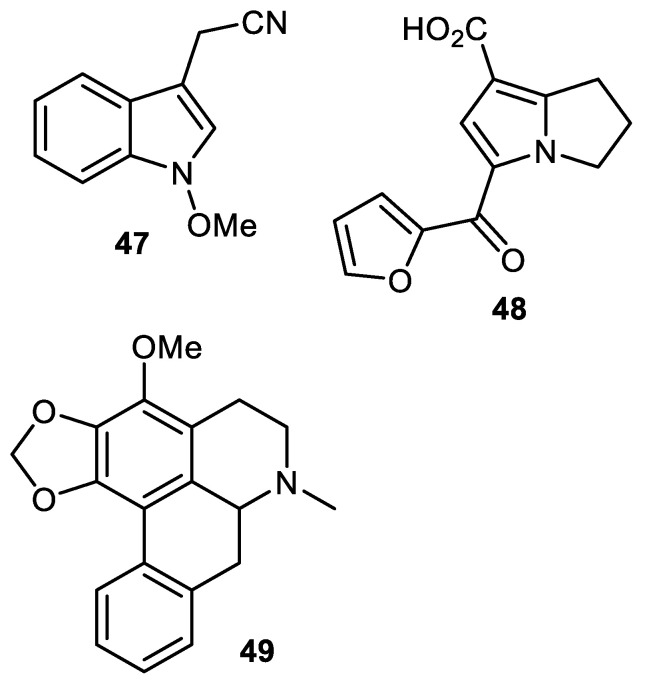
Structures of alkaloids **47**–**49**.

Caulilexin C (**47**) (Figure 7) was reported from the peel of *Elaeagnus rhamnoides* and this metabolite is included in the family of the phytoanticipins. Indol glucosinolates are the biosynthetic precursors of caulilexins [39]. Previously, this compound was reported from the cauliflower [48] as well as from *Brassica rapa* [49]. In another report, the pyrrolizine alkaloid named punicagranine (**48**) was reported from the peels of *Punica granatum* (pomegranate) and its structure was confirmed through an X-ray analysis. Punicagranine (**48**) is a very unusual pyrrolizine-type alkaloid featuring both a furan-2-carbonyl and carboxylic acid. Biosynthetically, this metabolite may be considered to be constructed through the condensation of the pyrrolizine alkaloid and 2-furoic acid. In a biological assay, punicagranine (**48**) exerted anti-inflammatory effects with an IC_50_ of 22.8 μM while the same compound did not show cytotoxicity towards RAW 264.7 cells [50]. 

Justino et al. [51] demonstrated that the polyphenol-enriched fraction of the fruit peel of *Annona crassiflora* illustrated promising antioxidant effects and these fractions can be employed in clinical applications to treat diabetes complications. Further study indicated that ca. 15 mg kg^−1^ day^−1^ could be considered to be an initial dose to undertake all important human studies. Stephalagine (**49**) was isolated from the fruit peel of *A. crassiflora*. The EtOH extract of *A. crassiflora* peels inhibited pancreatic lipase (PL) with IC_50_: 104.5 µg/mL and notably the stephalagine showed higher PL inhibition with IC_50_: 8.35 µg/mL [52]. In addition, this alkaloid exerted significant antinociceptive effects in vivo [53].

**Figure 8:**
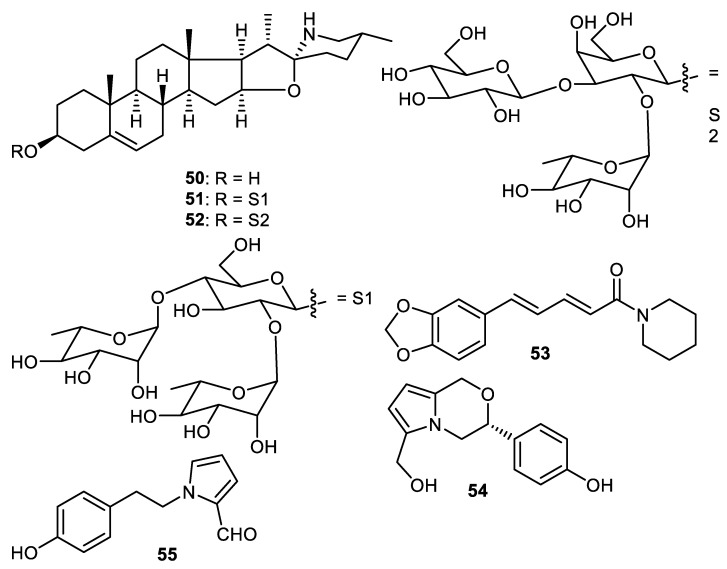
Structures of alkaloids **50**–**55**.

Steroidal alkaloids named solasodine (**50**), solamargine (**51**) and solasonine (**52**) (Figure 8) were isolated from the fruit peels of *Solanum melongena*. Alkaloids **50–52** exerted cytotoxic effects towards HCT116 (colon cancer), HEPG2 (liver cancer), HEP2 (larynx cancer), HELA (cervix cancer) and MCF7 (breast cancer), with IC_50_ values ranging from 2.1 to 9.0 µM [54]. Further study revealed that alkaloids **50–52** induced potent cytotoxic effects towards two liver cancer cells (HepG2 and Huh7) which were associated with S-phase cell cycle arrest. In addition, these molecules induced potent apoptosis in Huh7 cells [55].

Piperine (**53**) was isolated form the *Punica granatum* fruit peel and inhibited cathepsin D protease with IC_50_: 12.0 µg/mL [56]. The pyrrole alkaloid, strychnuxin (**54**) was reported from *Strychnos nuxblanda* fruit peels and showed no effect on α-glucosidase enzyme [57]. A potent elicitor, reticine A (**55**), was isolated from the fruit peel of *C. reticulate*. and the in vivo experiments of this alkaloid suggested it had significant control effects and this molecule was more potent than the elicitor benzothiadiazole (standard compound) [35].

**Figure 9:**
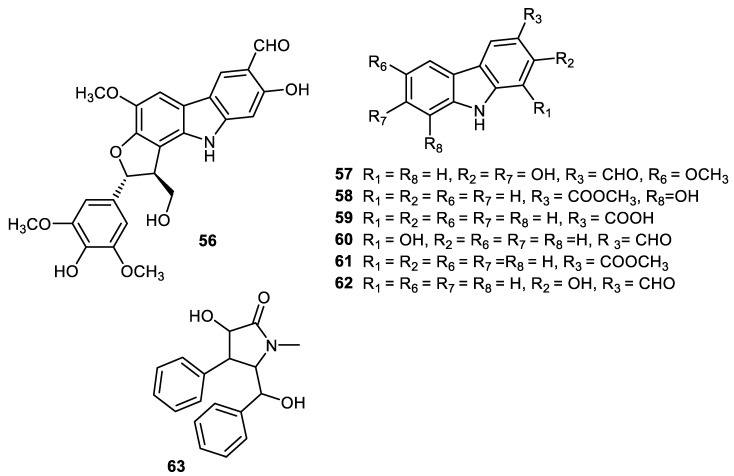
Structures of alkaloids **56**–**63**.

Carbazole alkaloids, claulansine K (**56**), claulansine J (**57**), carbazole-3-carboxylic acid (**58**), methyl 8-hydroxycarbazole-3-carboxylate (**59**), methyl carbazole-3-carboxylate (**60**), O-demethylmurrayanine (**61**) and mukonal (**62**) (Figure 9) were produced by peels of *Clausena lansium*. Alkaloid **56** illustrated α-glucosidase effects with the IC_50_: 0.11 mM. On the other hand, alkaloid **57** demonstrated moderate antibacterial potential towards *Staphylococcus aureus* [58]. Another alkaloid, clausenamide (**63**), was isolated from peels of *Clausena lansium* and illustrated α-glucosidase effects [37]. 

## 8. Benzoyltyramines

**Figure 10:**
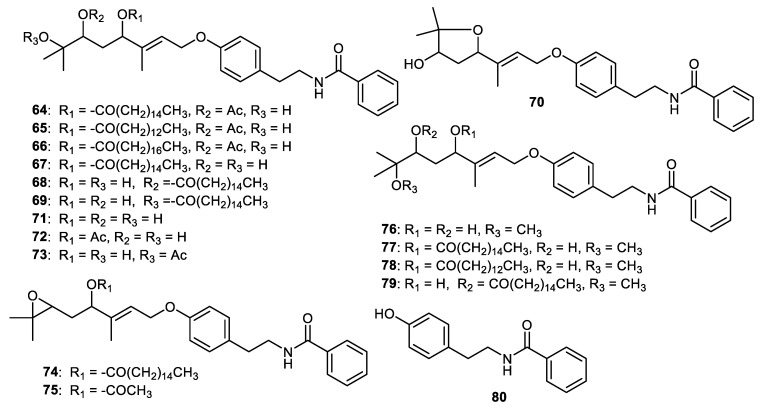
Structures of alkaloids **64**–**80**.

Benzoyltyramines, atalantums A-G (**64–70**) along with molecules **71–75** (Figure 10) were isolated from *Atalantia monophylla* fruit peels. Among these benzoyltyramines, atalantum (**64**) displayed the strongest cytotoxic effects towards cholangiocarcinoma cells (KKU-M156) with IC_50_: 1.9 μM. Notably, this compound was 4.7-fold more active than the standard ellipticine (IC_50_: 9.9 μM). On the other hand, atalantum A (**64**) demonstrated potent cytotoxic effects on KKU-M214 cells (IC_50_: 3.0 μM) and this activity was slightly higher than another standard 5-fluorouracil (IC_50_: 3.76 μM). In addition, benzoyltyramines **64** and **70** exhibited cytotoxicity towards KKU-M214 cells, with IC_50_: 8.44 and 7.37 μM, respectively. In addition, molecules **65**, **67** and **74** displayed significant effects on KKU-M213 cells with IC_50_: 2.3, 5.6 and 2.7 μM, respectively. The effects of these compounds were higher than ellipticine (IC_50_: 6.5 μM). In contrast, molecule **66**, which featured the longest hydrocarbon chain, exerted weak cytotoxic effects (IC_50_: 12–29 μM) [59]. 

Among the diols **67–69**, molecule **67** possessed potent cytotoxic effects toward the KKU-M156 and KKU-M213 cells (IC_50_: 2.8 and 5.6 μM) while molecule **68** displayed significant effects on KKU-M156 and KKU-M214 (IC_50_: 1.9 and 8.4 μM, respectively). On the other hand, compound **69** demonstrated weak effects towards three cancer cells (IC_50_: 16.1–31.4 μM) and these findings indicated that the palmitoyloxy group’s regiochemistry plays a crucial role. Concerning molecules **64** and **67**, the authors proposed that the C-6 acetate group enhances the cytotoxic effects ca. 9-fold toward the KKU-M214 cells. In addition, the 6,7-dihydroxy analog **67** displayed ca. 7- and 11-times stronger effects compared to the corresponding 6-acetoxy analog (compound **64**) towards KKU-M213 and KKU-M156 cells, respectively. The cytotoxic effects of compound **68** displayed a 1.42-fold stronger effect towards KKU-M156 cells than compound **67**. Interestingly, diols **67** and **68** were selective towards KKU-M156 cells, whereas diol **69** exerted weaker effects. The above findings suggested that the relative position of the hydroxy groups could play a critical role in the degree of cytotoxicity. Comparing the cytotoxic effects of compounds **74** (IC_50_: 2.7 μM) and **75** (IC_50_: 11.1 μM), **74** exhibited much better effects towards the KKU-M213 cell line, suggesting that the C-4 palmitoyloxy group better enhanced the effects compared to the acetoxy group [59]. 

Benzoyltyramines, atalantums H-K (**76–79**) along with atalantums D-G (**67–70**) and **80** were reported form the fruit peels of *Atalantia monophylla*. Biological studies showed that molecules **67** and **68** exerted cytotoxic effects towards cervical (HeLa), breast (MCF-7) and colon (HCT116) cancer with IC_50s_: ranging from 16–25 and 15–18 µg/mL, respectively. On the other hand, benzoyltyramine **69** was slightly more active than compound **67** (IC_50s_: ranging from 16–25 µg/mL), whereas compound **70** was slightly less active with IC_50s_: ranging from 20–35 µg/mL [60]. 

## 9. Flavones, Flavanone and Condensed Tannins

**Figure 11:**
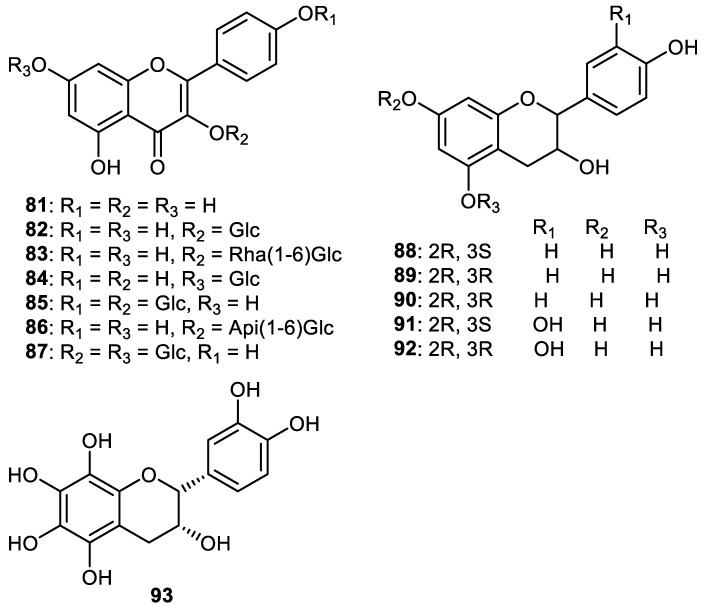
Structures of flavonoids **81**–**93**.

Flavones **81**–**93** (Figure 11) were isolated from the fruit peels of *Wisteria floribunda* and screened for PDGF-induced VSMC proliferation. Flavones **81**, **88**, **91** and **92** were the most promising compounds with IC_50s_: 3.6, 6.7, 4.3 and 4.6 µM, respectively. Of the flavones, **82–87**, **90** and **93** possessed moderate inhibition (IC_50s_: 19.3–39.5 μM). On the other hand, compound **89** was only weakly active (IC_50_ > 50 μM) [61]. 

**Figure 12:**
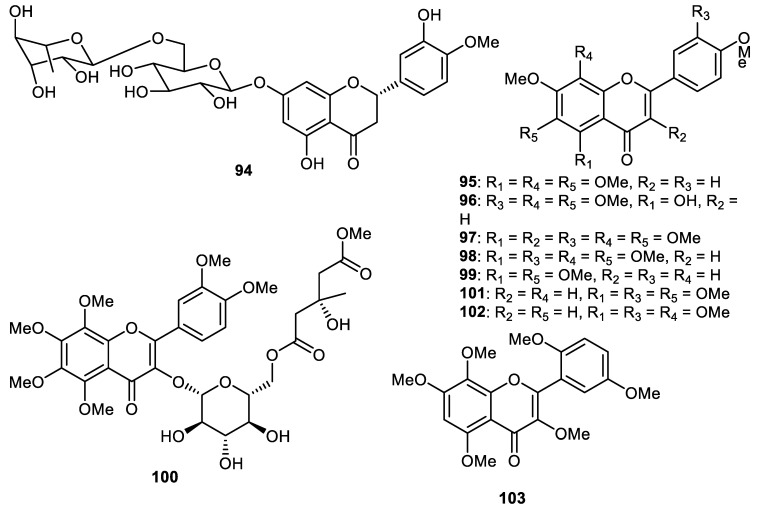
Structures of flavonoids **94**–**103**.

Hesperidin (**94**) (Figure 12) is largely present in citrus species such as *Citrus sinensis* (sweet orange peel) and tangerine *(C. reticulata)* [62,63]. In addition, hesperidin (**94**) and naringin are present in orange juice and these natural products are reported to be present in human plasma after diets involving grapefruit and orange as food sources. Hesperidin (**94**) exerted potential anti-inflammatory, anticarcinogenic, antimicrobial and antioxidant effects [63] and has additionally been successfully employed as a supplemental dietary agent since it has been found that a deficiency thereof causes weakness, aches and night leg cramps. Supplemental hesperidin has been used to treat excess swelling of the legs and oedema [63]. 

Hesperidin (**94**) along with the polymethoxy flavones **95–98** were isolated from the fruit peels of *Citrus* ‘Hebesu’ and tested for their biological effects. Flavones **96–98** exerted potent anti-neuroinflammatory effects through the inhibition of the expression of IL-1β mRNA [20]. Notably, polymethoxyflavonoids (PMFs) are found to be largely present in citrus plants. It is thus not surprising that citrus peels are one of the richest sources of PMFs including *C. sinensis* (sweet orange) and *C. reticulata* (mandarin) [64]. PMFs have demonstrated a plethora of biological effects viz., anticancer, antioxidative, antiviral and anti-inflammatory effects [64,65,66,67]. 

Tangeretin (**95**), in anti-arenaviral screening, demonstrated a reduction (65%) in pseudotype infectivity with EC50: 6.0 µM [53]. LASV-GP/HIV-luc infectivity was also decreased by tangeretin (**95**) in different cell lines, such as U-87 MG cells (glioblastoma), A549 cells (lung cancer), Vero E6 cells (African green monkey kidney cell), with EC_50_: 4.5, 8.8 and 2.7 μM, respectively [27]. Tangeretin (**95**) also had significant effects against VSV with an EC50 of 0.72 μM. Further study demonstrated that tangeretin (**95**) with an EC_50_ of 0.26 μM, exerted a significant inhibitory effect on LASV infection. In addition, flavone **95** prevented LASV-GP/HIV-luc infectivity along with pseudotype viruses such as LUJV, WWAV, SABV GTOV, MACV, CHPV and JUNV with EC_50_: ranging from 2.3 μM to 11.4 μM [27,68].

Nobiletin (**98**), isolated from citrus fruit peel, demonstrated antioxidant potentials. In addition, this flavone is also isolated from *C. reticulate* (mandarin oranges), *C. sinesis* (sweet oranges), *C. miaray* (Miaray mandarins) [69], *C. depressa* (flat lemons) [70,71], *C. tangerine* (tangerines), *C. aurantium* (bitter oranges) [72], *C. unshiu* (Satsuma mandarins) [73,74], *C. reshni* (Cleopatra mandarins) [75], *C. tachibana*, *C. leiocarpa* (Koji Oranges), *C. tardiva* (Natsu Mikans), *C. succosa* (Jimikan), *C. Kinokuni* (Kinokuni Mandarins), *C. erythrosa* (Fukushu) and *C. sunki* (Sunkat) [76]. 

Zhang et al. [62] demonstrated in vivo studies on the protective effects of flavone **98** in the mediation of cardiac hypertrophy in which it was demonstrated to inhibit aortic banding (AB)-induced cardiac hypertrophy, as measured by cardiomyocytes cross-sectional area, cardiac weight-to-body weight ratio, cardiac function and through gene expression of markers of hypertrophy. The **98** supplementation in cardiac hypertrophy inhibited NOX4 and NAPDH oxidase (NOX)2 expression and alleviated myocyte apoptosis and endoplasmic reticulum (ER) stress. Further studies suggested that the administration of flavone **98** decreased cardiomyocyte hypertrophic response in neonatal rat as stimulated through phenylephrine (PE) and decreased ER stress. However, this study indicated that **98** significantly reduced NOX2 expression but did not affect NOX4 expression in vitro. The authors suggested that the inhibition of oxidative and ER stress by flavone **98** in the cardiac muscle may indicate an effective therapy for the management of cardiac hypertrophy [77]. 

Nobiletin (**98**) has additionally been reported to possess potential anti-tumor and anti-inflammatory potential [78,79]. In addition, this flavone has been reported to inhibit NF-κB activation mediated by LPS in macrophages in mice [80]. However, it is still unknown how nobiletin (**98**) prevents the activation of NF-κB. It is however known that it reduces the activation of NF-κB by inhibiting its DNA-binding potential. In addition, flavone **98** blunted NF-κB transactivation and DNA-binding capacity of p50/p65 and was mediated by LPS [78]. Additional results demonstrated that it neither altered LPS-mediated phosphorylation and IκBα degradation, nor the NF-κB translocation to the nucleus. These findings, therefore, show that the inhibitory action of nobiletin (**98**) on proinflammatory mediator’s expression may involve the inhibition of NF-κB activity [78]. 

**Figure 13:**
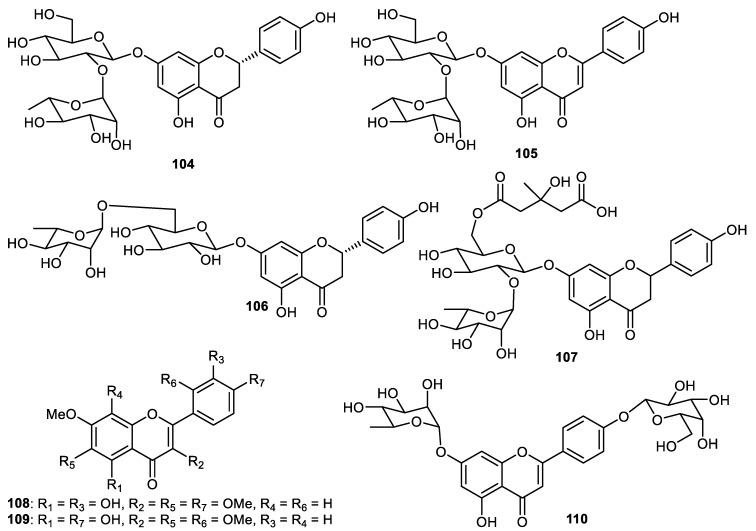
Structures of flavonoids **104**–**110**.

Nobiletin (**98**) exerted cytotoxic effects towards HT-29 with IC_50_: 4.7 µM [81]. This flavone showed protective effects towards a variety of cancers in in vivo studies [73,82,83,84,85,86,87]. Phytochemical investigations of the peels of *C. unshiu* (Hallabong) resulted in the isolation and purification of tetramethyl-O-scutellarin (**99**) and this material demonstrated anti-inflammatory effects with IC_50_: 57.4 μM. Further study revealed that this flavone inhibited the generation of proinflammatory mediators such as IL-1β, TNF-α, PGE2 and IL-6 [28]. Polymethoxylated flavonol glucoside derivatives, citrusunshitin A (**100**), along with flavones **101**–**103** were produced by the fruit peels of *C. reticulate* [88]. In another report, the flavones naringin (**104**), rhoifolin (**105**), naringenin 7-rutinoside (**106**), melitidin (**107**), vitexicarpin (**108**), chrysosplin (**109**) and rubranonoside (**110**) (Figure 13) were isolated from the peels of *C. grandis* [34].

**Figure 14:**
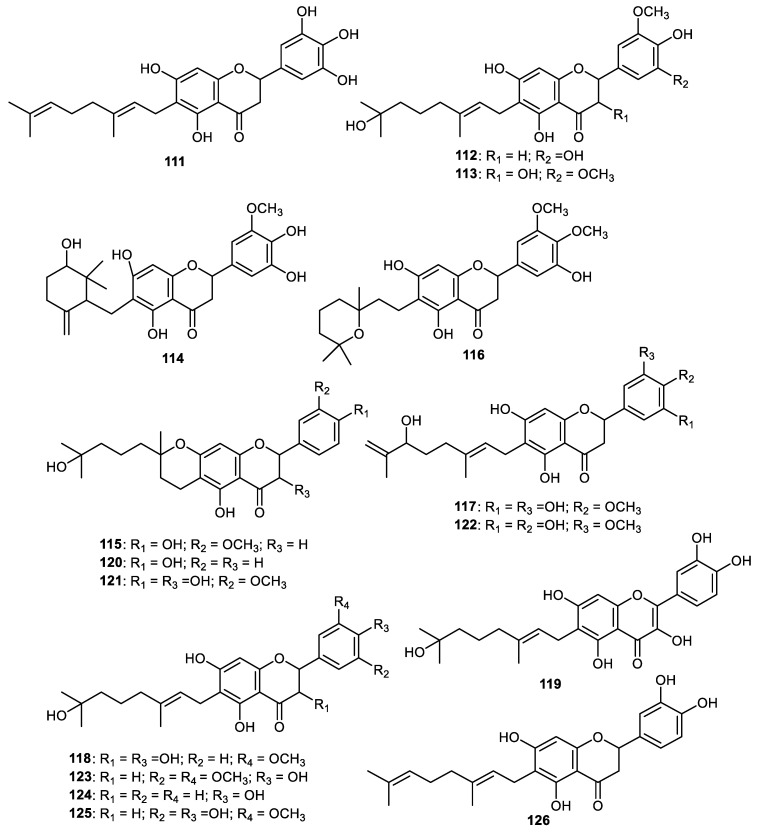
Structures of flavonoids **111**–**126**.

Paucatalinones C–E (**111–113**) (Figure 14), representing geranylated flavonoids, are produced by the fruit peel of *Paulownia catalpifolia*. It was found that these flavonoids exerted good antioxidant effects [89]. Paucatalinones F-K (**114**–**119**) along with the geranylated flavanones **120**–**126** are produced by the *Paulownia catalpifolia* fruit peel [90]. Notably, paucatalinone F (**114**) featured an oxygenated cyclogeranyl group while interestingly, paucatalinone H (**116**) contains a pyranoidal geranyl group. These flavonoids were screened for their protective potential on human umbilical vein endothelial cells (HUVECs) injury. The flavones **117**, **122** and **125** exerted proliferative effects in a dose-dependent way from 1 to 50 μmol/L. On the other hand, metabolites **114**, **115** and **123** at 50 μmol/L and flavones **126** at 20 and 50 μmol/L decreased their proliferative effects. These results indicated that the above flavonoids demonstrated their cytotoxic effects to HUVECs at these concentrations and flavones **116**, **118** and **119** exerted better proliferative effects at only ≥20 μmol/L concentrations. Interestingly, flavonoids **114** possessed antiapoptotic effects [90]. 

**Figure 15:**
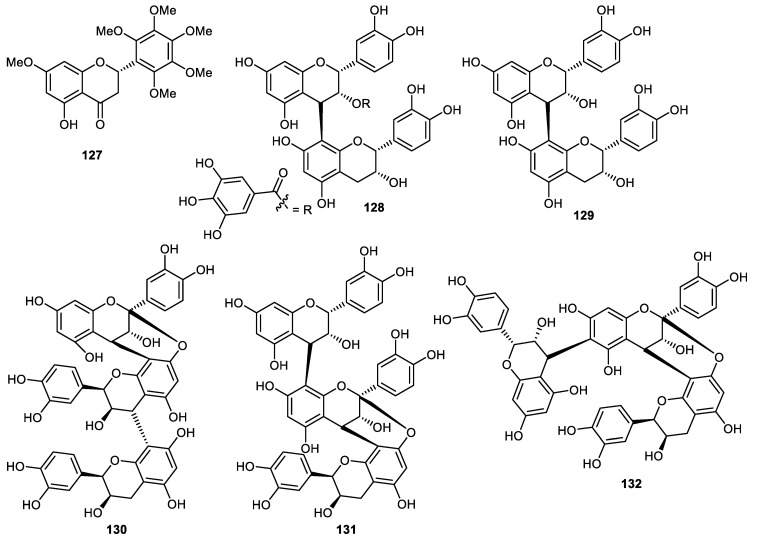
Structures of flavonoids **127**–**132**.

Flavanone **127** (Figure 15) was isolated from *Citrus reticulata* fruit peels [91]. It is well known that proanthocyanidins are produced by fruits, beans and nuts [92]. The proanthocyanidin dimers (−)-epicatechin gallate-(4β→8)-(−)-epicatechin (**128**), procyanidin B2 (**129**), have been reported from *Pyrus pyrifolia* (pear) fruit peels [93,94,95]. Pear fruit peels also produced cinnamtannins B1 (**130**), aesculitannin A (**131**) and (−)-epicatechin-(4β→6)-(−)-epicatechin-(4β→8, 2β→O→7)-(−)-epicatechin (**132**) [95] and these proanthocyanidin trimers displayed anticancer, antibacterial and antioxidative effects [96,97,98,99].

**Figure 16:**
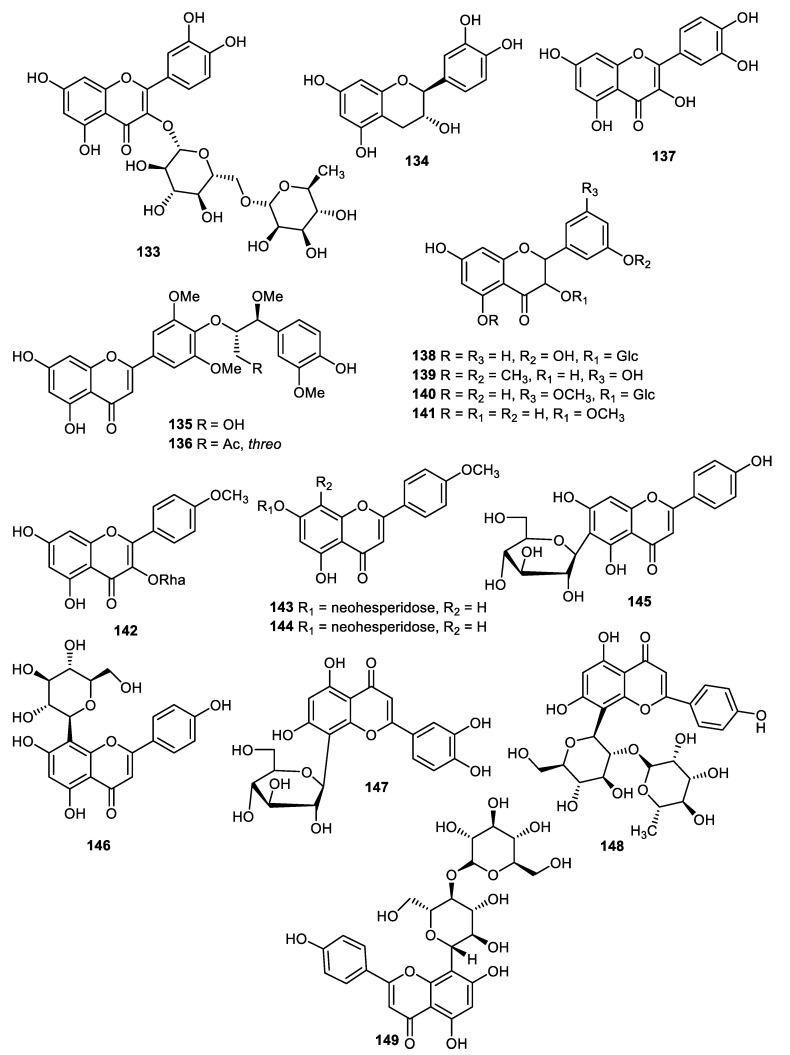
Structures of flavonoids **133**–**149**.

The pear (*Pyrus ussuriensis*) peels produced flavones, including rutin (**133**), (−)-catechin (**134**), tricin 4′-O-[threo-β-guaiacyl-(7″-O-methyl)-glyceryl] ether (**135**), tricin 4′-O-[threo-β-guaiacyl-(7″-O-methyl-9″-O-acetyl)-glyceryl] ether (**136**) and quercetin (**137**) (Figure 16). The flavones **135** and **136** illustrated antioxidant effects with IC_50_: 43.5 and 39.1 µg/mL, respectively [100]. Astragalin (**138**), quercetin 5,4′-dimethyl ether (**139**), isorhamnetin-3-O-glucoside (**140**) and isorhamnetin (**141**) were produced by *Opuntia ficus-indica* peels and flavone **139** demonstrated significant antibacterial effects towards *Streptococcus pneumoniae, Stenotrophomonas maltophilia, Moraxella catarrhalis, Klebsiella pneumoniae, Pseudomonas aeruginosa and Legionella pneumophila*. In addition, flavone **140** illustrated good antibacterial potential towards *Streptococcus pneumoniae, Stenotrophomonas maltophilia, Moraxella catarrhalis, Klebsiella pneumoniae* and *Pseudomonas aeruginosa* [101]. 

Kaempferide-3-*O*-rhamnopyranoside (**142**), acacetin-7-*O*-neohesperidoside (**143**) and acacetin-7-*C*-neohesperidoside (**144**) were reported from fruit peels of *Fortunella japonica* [42]. It was found that isovitexin (**145**), vitexin (**146**), orientin (**147**), vitexin-2″-O-rhamnoside (**148**) and vitexin-4″-O-glucoside (**149**) were reported from *Benincasae exocarpium* fruit peels and screened for antidiabetic effects. Flavones **145–149** inhibit AGE better than AMG (IC_50_: 178.8 μM, positive control) with IC_50_: 20.2, 19.5, 9.6, 29.5 and 14.5 μM, respectively. In addition, flavones **145–149** inhibit the α-glucosidase enzyme better than the standard acarbose (IC_50_: 49.4 μM, except flavone **149**) with IC_50_: 48.1, 52.2, 36.1, 12.8 and 65.3 μM, respectively [102]. 

## 10. Lignans

**Figure 17:**
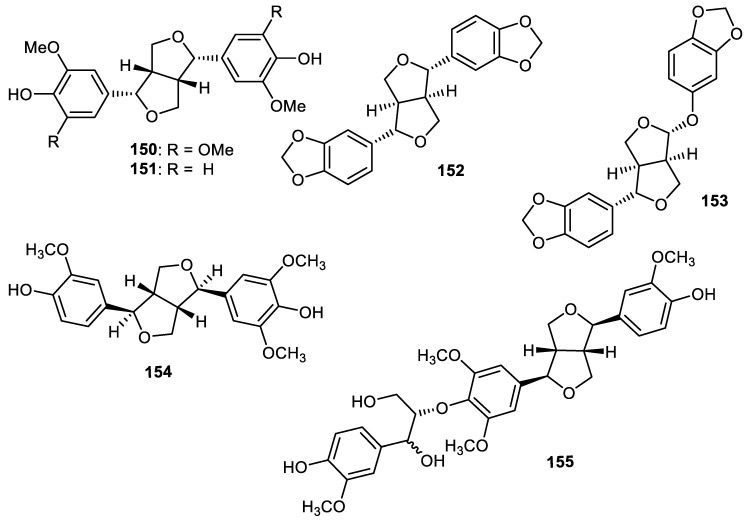
Structures of *lignans* **150**–**155**.

Two lignans named syringaresinol (**150**) and (−)-pinoresinol (**151**) (Figure 17) were reported from the fruit peels of *Wisteria floribunda* and exerted PDGF-induced VSMC proliferation with IC_50_: > 50 and 46.6 µM, respectively [61]. Sesamin (**152**) and sesamolin (**153**) were produced by *Strychnos nuxblanda* fruit peels. Notably, metabolites **152** and **153** exhibited *α*-glucosidase with IC_50_: 447 and 215 μM, respectively, while the activities of these same lignans were higher than acarbose (standard compound; IC_50_: 526 μM). On the other hand, these compounds were not active as acetylcholinesterase enzymes [57]. Furofuran-type lignans named pinoresinol (**154**) and medioresinol (**155**) were isolated from *Annona squamosa* fruit peels and both compounds illustrated anti-inflammatory effects with IC_50_: 45.4 and 6.2 µM, respectively [45]. 

## 11. Hydrolyzable Tannins 

**Figure 18:**
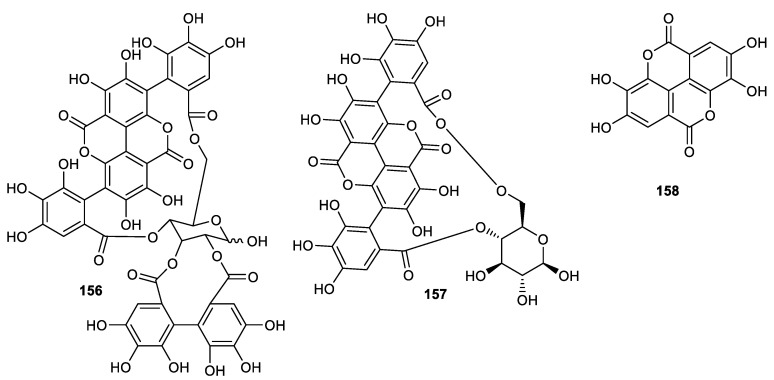
Structures of *tannins* **156**–**158**.

Punicalagin (**156**) (Figure 18) was isolated from the peel of the pomegranate and exerted antifungal effects towards *Trichophyton rubrum* with an MIC of 62.5 μg/mL, whereas the same compound was cytotoxic on Vero cell (90%) [103]. Punicalagin (**156**), punicalin (**157**) and ellagic acid (**158**) were isolated from pomegranate fruit peels. Tannins **156–158** prevent protease-mediated effects in vitro through acting on HCV NS3/4A protease directly (with IC_50_: < 0.1 µM for tannins **156** and **157** and compound **158** has IC_50_: 1.0 µM). Notably, these compounds are all reported to be associated with no toxic effects side effects ex vivo and were quite safe at 5000 mg/kg when given acutely in BALB/c mice. Pharmacokinetics data indicated that compounds **156–158** are readily bioavailable [104]. 

Literature revealed that tannins can form complexes with metal ions as well as target macromolecular polysaccharides and proteins [105]. Hence, these compounds may interact with enzymes through their interaction with any zinc moiety thus preventing its activity. In addition, some studies have significantly proven their selective binding of PGN, PLN and EA to NS3 protease enzymes at their substrate binding sites and this is further supported by molecular docking studies [23,104]. It is noted that molecules **156** and **157** possess galloyl residues that provide further evidence for their inhibitory effects against NS3/4A protease. 

Further study revealed that compounds **156–158** demonstrated biological effects towards RNA replication of HCV and the exact mechanism of this activity is yet to be investigated. Since compounds **156**–**158** inhibit NS3 protease, thus affecting HCV polyprotein proteolytic processing which in turn leads to a decreased active viral RNA-dependent RNA polymerase level. This eventually causes a decreasing level of HCV RNA [104]. Of note is that tannin **158** results in apoptosis in the cell line of human prostatic cancer by decreasing antiapoptotic factors including HuR (human antigen R), HO-1 (Heme oxygenase-1) and SIRT1 (silent information regulator). It also decreases apoptotic markers including IL-6 (interleukin-6) and TGF-β (transforming growth factor -β) in cell line of prostatic cancer [106]. Compound **158** also mediates the apoptotic process in cancer cells of the pancreas through inhibiting NF-kB and the mitochondrial depolarization process [107] and demonstrated an anticancer effect through decreasing the expression of genes involved in oxidative stress [108].

## 12. Phloroglucinol

**Figure 19:**
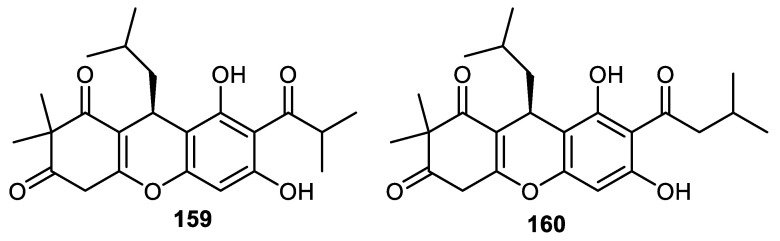
Structures of phloroglucinol **159** and **160**.

Myrciarone A (**159**) and rhodomyrtone (**160**) (Figure 19) are produced by the fruit peels of *Myrciaria dubia* and were screened for their antimicrobial effects where it was found that these compounds displayed potent antimicrobial effects towards *Bacillus subtilis* and *B. cereus* with MIC: 1.56 and 0.78 µg/mL, respectively. Notably, the activities of metabolite **159** against *B. subtilis* and *Streptococcus aureus* were equal to the standard kanamycin, whereas the effects of the same compound against *B. cereus* and *Micrococcus luteus* were 4-fold higher than the standard kanamycin (MIC: 6.25 µg/mL) [109]. 

The activities of rhodomyrtone (**160**) towards *B. cereus* and *M. luteus* (MIC: 0.78 µg/mL) were 8-fold higher than kanamycin (MIC: 6.25 µg/mL). The antimicrobial effects of molecule **160** against *S. mutans* (MIC: 1.56 µg/mL) were equal to the standard kanamycin, whereas the same compound was 2-fold more active towards *S. aureus* (MIC: 0.78 µg/mL) and *S. epidermidis.* Myrciarone A (**159**) displayed the same level of activities against *S. epidermidis* and *S. mutans* with MIC: 3.13 µg/mL and its effects were 2-fold less than kanamycin (MIC: 1.56 µg/mL). Metabolites **159** and **160** were unfortunately not active towards *Escherichia coli*, *Salmonella typhimurium*, *Pseudomonas aeruginosa*, *Candida albicans* and *Saccharomyces cerevisiae* [109]. 

## 13. Triterpenoids

### 13.1. Onocerane Triterpenoids

**Figure 20:**
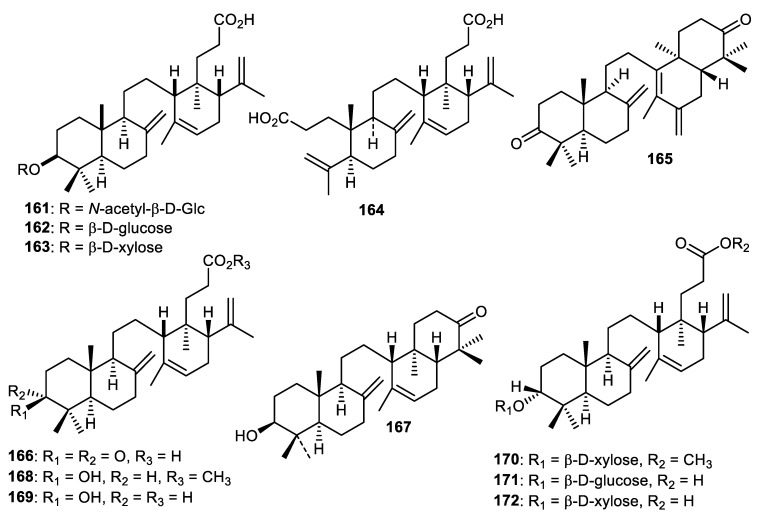
Structures of onocerane triterpenoids **161**–**172**.

The fruit peel of *Lansium domesticum* produced *onocerane* triterpenoids named lansiosides A-C (**161–163**) (Figure 20) along with lansic acid (**164**) and all were effectively shown to decrease leukotriene D4-mediated contraction of guinea pig ileum [110]. In another report, lamesticumin G (**165**), lansionic acid (**166**), 3β-hydroxyonocera-8(26),14-dien-21-one (**167**), compound **168** and lansiolic acid (**169**) were isolated from the fruit peels of *Lansium parasiticum*. Lamesticumin G (**165**) demonstrated α-glucosidase inhibition with IC_50_: 2.27 mM [111]. In another report, methyl lansioside C (**170**), lansiosides B (**171**) and C (**172**) were reported from the fruit peels of *Lansium parasiticum*. Triterpenoids **165** (SC_50_: 14.5 mM) and **171** (SC_50_: 13.7 mM) exerted moderate antioxidant effects, whereas triterpenoid **172** possessed weaker antioxidant effects (SC_50_: 23.6 mM) while compounds **170–172** were not active in α-glucosidase screening [112]. 

**Figure 21:**
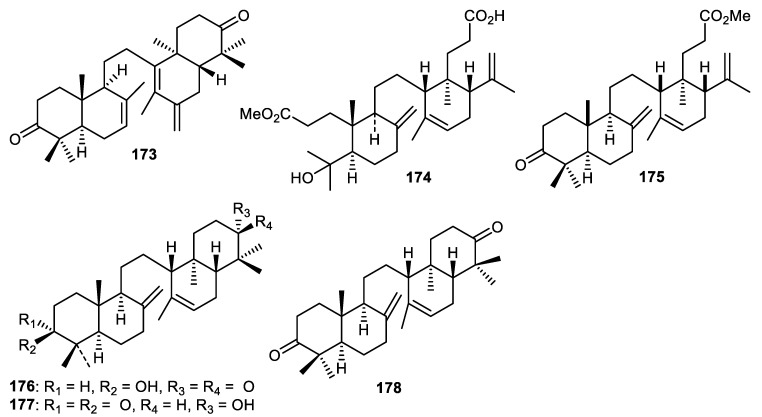
Structures of onocerane triterpenoids **173**–**178**.

Phytochemical investigation of *Lansium domesticum* fruit peels resulted in the isolation of an onoceranoid named onoceradienedione (**173**) (Figure 21) which displayed cytotoxic effects towards T47D, HeLa and A459 cells with IC_50_: 30.6, 32.3 and 13.7 µg/mL, respectively [113]. In another report, the same fruit peels produced another onoceranoid triterpenoid named lamesticumin A (**174**) which possessed cytotoxic effects towards epithelial breast cancer (T47D) with IC_50_: 15.6 μg/mL [114]. Tanaka et al. [115] also investigated *L. domesticum* fruit peels and reported finding onoceranoid triterpenoids **175–177** all of which displayed moderate toxicity towards *Artemia salina*. The molecule described as α,γ-onoceradienedione (**178**) was produced by *Lansium domesticum* fruit peels and possessed moderate effects towards *C. albicans* and *A. niger* [116].

### 13.2. Miscellaneous Triterpenoids

**Figure 22:**
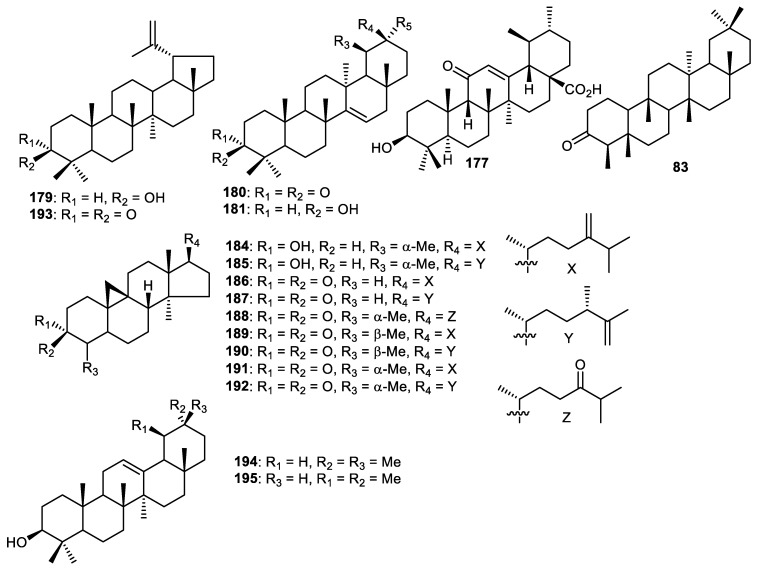
Structures of triterpenoids **179**–**195**.

Lupeol (**179**), taraxerone (**180**) and taraxerol (**181**) (Figure 22) were reported from the fruit peels of *Wisteria floribunda* and were shown to exert PDGF-induced VSMC proliferation with IC_50_: 5.4, 7.5 and >50 µM, respectively [61]. Fruit peels of *Punica granatum* produced ursolic acid (**182**) which was demonstrated to inhibit cathepsin D with IC_50_: 8.3 µg/mL [56]. Friedelin (**183**) was isolated from *C. reticulata* fruit peels [35]. Cycloartane triterpenes **184**–**188** [117] and **189**–**192** [118] were produced by *Musa sapientum* fruit peels (banana). Lupenone (**193**), β-amyrin (**194**) and α-amyrin (**195**) were obtained from fruit peels of *Fortunella japonica* [42].

## 14. Steroids

**Figure 23:**
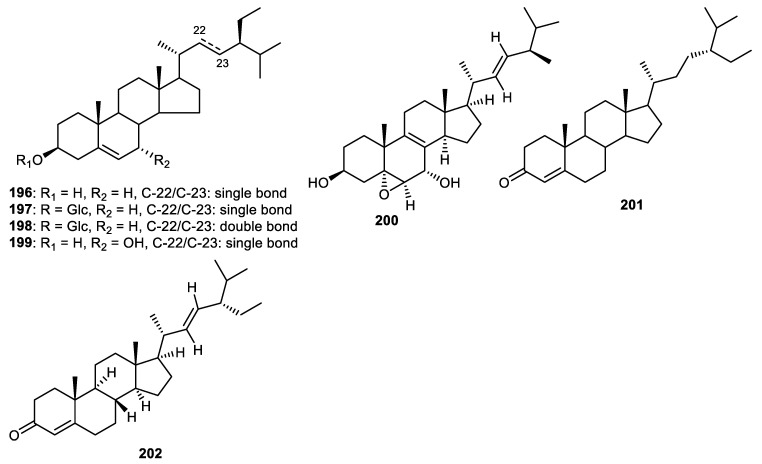
Structures of steroids **196**–**202**.

Fruit peels of *Wisteria floribunda* produced β-sitosterol (**196**) and β-sitosterol glucopyranoside (**197**) (Figure 23) and exerted PDGF-induced VSMC proliferation with IC_50_: 19.8 and >50 µM, respectively [46]. The steroid **197** and β-stigmasterol-3-O-β-D-glucoside (**198**) were produced by the fruit peels of *Solanum melongena* [54], whereas steroid **199** was isolated from *C. reticulata* fruit peels [35]. Steroids **197** and **198** demonstrated cytotoxic effects towards HCT116 (colon cancer), HEP2 (larynx cancer), MCF7 (breast cancer), HEPG2 (liver cancer) and HELA (cervix cancer) with IC_50_ ranging from 2.2 to 13.4 µM [54]. In another report, steroids **200**–**202** were reported from the fruit peel of *Annona squamosal* and illustrated anti-inflammatory effects with IC_50_: 5.0, 5.2 and 5.4 µM, respectively [45]. 

## 15. Peptides

**Figure 24:**
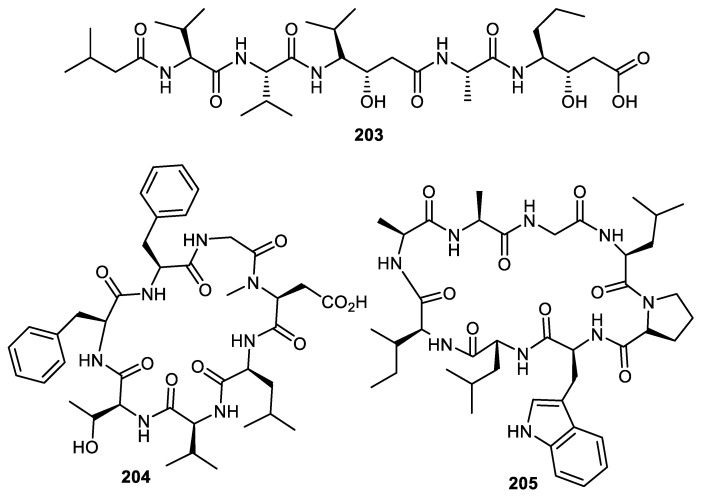
Structures of peptides **203**–**205**.

Pepstatin A (**203**) (Figure 24) was reported from *Punica granatum* fruit peel [56]. This peptide is a potent inhibitor of aspartic proteases (AP) with an inhibition constant (Ki) of 0.1 nM. Notably, semisynthetic derivatives of pepstatin have led to the discovery of potent AP inhibitors. Research showed that the statyl part of this peptide is thought to be responsible for the pepsin inhibition [119]. Two cyclic peptides **204** and **205** produced by the fruit peels of *C. medica* had their structures established via extensive spectroscopic techniques [120].

## 16. Miscellaneous

**Figure 25:**
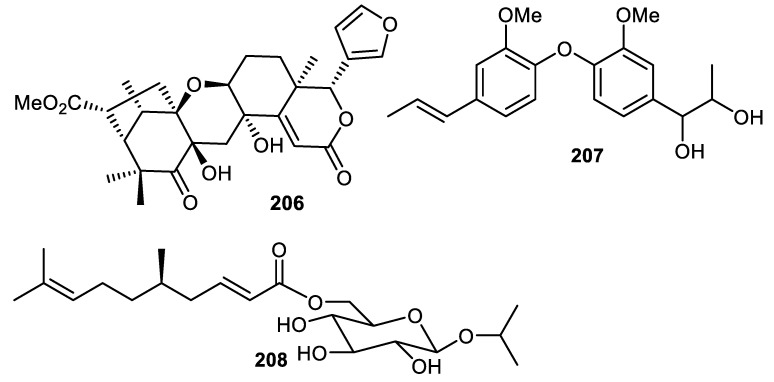
Structures of compounds **206**–**208**.

The tetranortriterpenoid, kokosanolide D (**206**) (Figure 25) was reported from *Lansium domesticum* fruit peels [121]. Biphenyl ether **207** was isolated from the fruit peel of *Elaeagnus rhamnoides*. In antiviral screening, compound **207** was found to cause a 3.49 log_10_ reduction in HSV-2 yield and this metabolite also possesses antiviral effects in the qPCR method [39]. Citrusoside A (**208**) was isolated from the peels of *Citrus hystrix* fruits and possesses butyrylcholinesterase inhibition activity with IC_50_: 376 µM [31]. 

**Figure 26:**
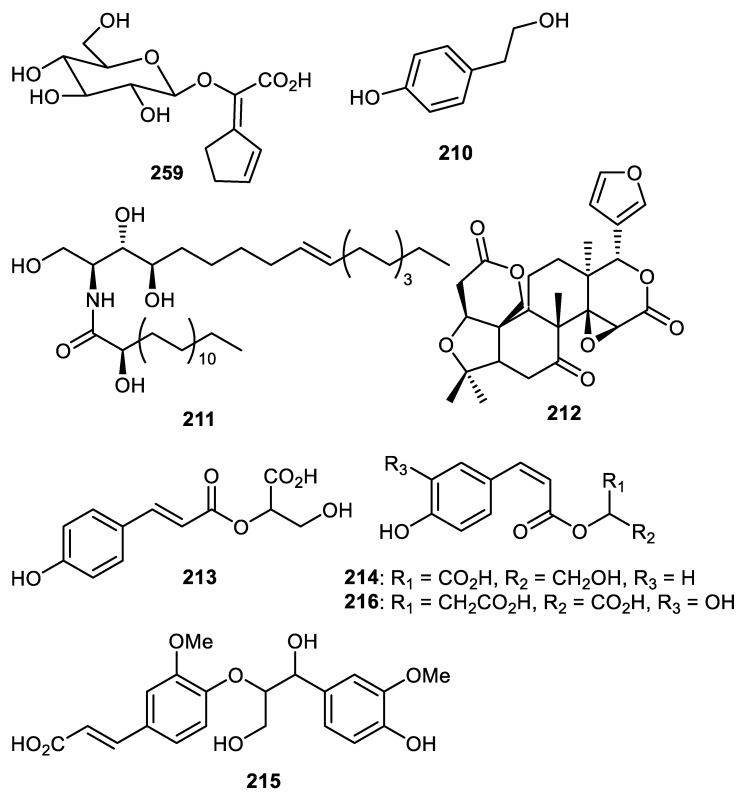
Structures of compounds **209**–**216**.

Passiflora edulis fruit peels produce edulilic acid (**209**) (Figure 26) that exhibited an antihypertensive effect [122]. *Strychnos nuxblanda* fruit peels produced tyrosol (**210**) which exhibited *α*-glucosidase effects with an IC_50_: 295 μM [57]. Compounds **211** and **212** were isolated from *C. reticulata* fruit peels [35]. Metabolites **213–216** were isolated from *Pyrus pyrifolia* fruit peels (pear) and **216** displayed higher antioxidant effects than caffeic acid (standard) [123]. 

**Figure 27:**
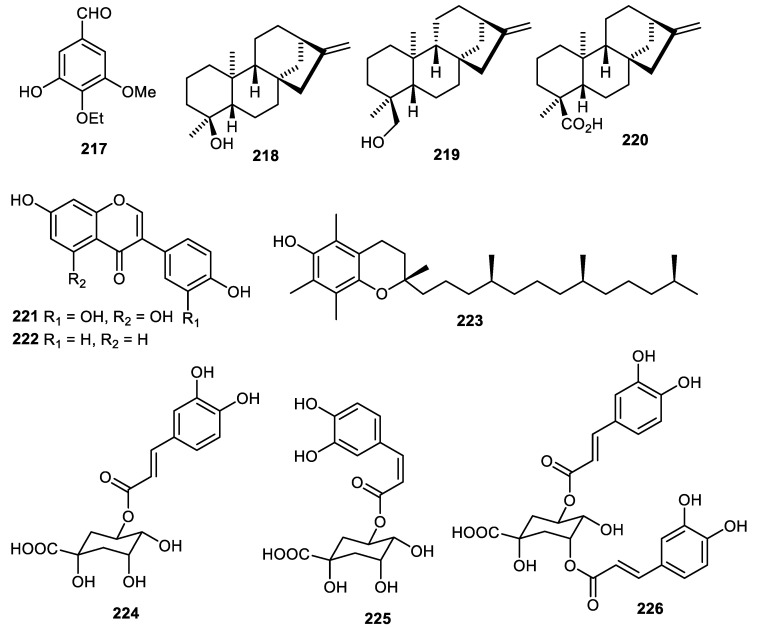
Structures of compounds **217**–**226**.

Benzaldehyde **217** (Figure 27) was produced by *C. reticulate* [36] while diterpenes **218–220** were produced by *Annona squamosal* fruit peel [45] and illustrated anti-inflammatory effects with IC_50_: 16.3, 19.7 and 38.5 µM, respectively [45]. The pear (*Pyrus ussuriensis*) peels produced orobol (**221**), daidzein (**222**) and possessed antioxidant effects with IC_50_: 29.1 and 88.5 μg/mL, respectively [100]. The *Fortunella japonica* fruit peels produced α-tocopherol (**223**) [42] while *Pyrus pyrifolia* (pear) fruit peels produced trans-chlorogenic acid (**224**), cis-chlorogenic acid (**225**) and 3,5-dicaffeoylquinic acid (**226**) [43].

**Figure 28:**
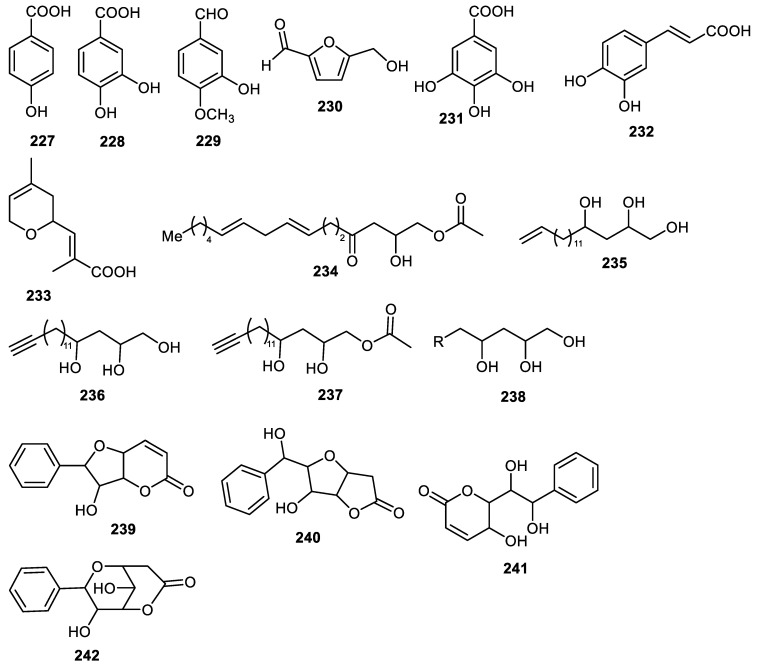
Structures of compounds **227**–**242**.

Phenolic compounds **227**–**232** (Figure 28) were isolated from *Benincasae exocarpium* peels and compounds **228**, **231** and **232** inhibited AGE with IC_50_: 79.7, 191.7 and 54.5 µM, respectively. On the other hand, molecules 228, 229 and 231 illustrated α-glucosidase inhibition with IC_50_: 48.6, 310.4 and 108.6 µM, respectively [102]. Monoterpenoid **233** was obtained from peels of *Clausena lansium* [37] while compounds **234–238** were reported from avocado fruit peels [124] and metabolites **239**–**242** were obtained from *Goniothalamus scortechinii* fruit peel [41]. 

## 17. Fruit Peels and Food Industries

A good number of countries have directed their industry to enhance their food supply sequence effectively because that will decrease food loss and waste. In order to counteract this issue, including active agents viz., antioxidant and antimicrobial molecules or extracts, into packaging materials is considered a feasible solution in order to increase food shelf life, decrease food losses and enhance the wealth of the food industry. Natural products, biopolymers and extracts are reported to maintain the safety and biocompatibility of the material addressing consumer health concerns. 

A great number of natural products have been isolated from fruit peels and these compounds illustrated antimicrobial, antioxidant and cytotoxic effects. In addition, Table 1 illustrates that many fruit peel extracts and essential oils possess antimicrobial and antioxidant effects [29,30,125,126,127,128,129,130,131,132,133,134,135,136,137,138,139,140,141,142,143,144,145,146,147,148,149,150,151,152,153,154,155,156,157,158,159,160,161,162,163]. Utilization of fruit peels can become a possible food industry, cosmetic industry and as a source for producing beneficial drugs. With a marked antibacterial potential, fruit-peel-derived natural products, extracts and essential oils could be utilized as antibacterial agents in food processing and storage. For instance, these peel-derived materials could suppress the spoilage bacteria, mainly *Pseudomonas antarctica*. These peel-derived materials revealed significant antibacterial effects towards pathogenic *Staphylococcus aureus* which causes food poisoning.

Antioxidants from natural sources are valuable bioactive compounds with well-demonstrated potentials for use in the food industry. Lipid oxidation along with microbial growth are the major cause of spoilage of foods, such as nuts, meats, fish, sauces, milk powders and oils. Fruit-peel-derived natural products, extracts and essential oils demonstrated significant antioxidant capacity and could be employed as a substitute for synthetic antioxidants to enhance the shelf life of foods. 

Pomegranate peel is reported to be a source of cellulose [164]. Cellulose is a biodegradable polymer and can be utilized in different food applications since it has been used in biomedical applications, such as carriers in drug delivery. Pectins are carbohydrate macromolecules present in citrus fruit peels, apple pomace and mango peels. Pectins are employed as an ingredient or additive for food in the preparation of jellies, jams and marmalades [164].

**Table 1:** Antimicrobial and antioxidant effects of fruit peel extracts and essential oils.

Fruit Peels	Antimicrobial and Antioxidant Effects	Ref.
Wampee (*Clausena lansium*)	**Antioxidant**: Extract(s) showed better effect than BHT	[29]
Red dragon fruit (*Hylocereus polyrhizus*) E	**Antibacterial**: *Staphylococcus aureus*; *Streptococcus mutans*; **Antifungal**: *Candida albicans;Aspergillus fumigatus* (EO); **Antioxidant**: E showed good effects	[125,126,127,128,129,130]
Melons (*Cucumis melo* L.) E	**Antibacterial**: *Staphylococcus epidermidis*; *Streptococcus pyogenes*	[130]
Passion *(Passiflora edulis*) E	**Antibacterial**: *Staphylococcus epidermidis*; *Streptococcus pyogenes*; **Antioxidant**: E showed good effects	[130,131]
Pineapple (*Ananas comosus*) E	**Antibacterial**: *Staphylococcus epidermidis*; *Streptococcus pyogenes*; *Pseudomonas aeruginosa*; **Antioxidant**: E showed good effects	[130,132]
*Carica papaya*	**Antibacterial**: *Pseudomonas aeruginosa*; **Antioxidant**: E showed good effects	[132]
Dragon Fruit *(Hylocereus undatus*) E	**Antibacterial**: *Staphylococcus epidermidis*; *Streptococcus pyogenes*	[130]
Watermelon (*Citrullus lanatus*) E	**Antibacterial**: *Staphylococcus epidermidis*; *Streptococcus pyogenes; Bacillus subtilis; Pseudomonas species; Staphylococcus aureus; Klebsiella pneumoniae; Protieus mirabilis*; **Antioxidant**: E showed significant effects	[130,133]
Mango (*Mangifera indica*) E	**Antibacterial**: *Staphylococcus epidermidis*; *Streptococcus pyogenes;***Antioxidant**: E showed significant effects	[130,134]
*Mangifera pajang*	**Antioxidant**: E showed significant effects	[135]
*Citrus reticulata*	**Antioxidant**: EO showed good effects; **Antibacterial**: *Escherichia coli; Staphylococcus aureus*; *Enterococcus faecalis*; *Salmonella typhi*; *Klebsiella pneumoniae*; *Pseudomonas aeruginosa;***Antifungal**: *Candida albicans*	[136,137]
Pomelo (*Citrus maxima*)	**Antioxidant**: E showed significant effects	[138]
*Citrus aurantifolia* (EO)	**Antibacterial**: *Streptococcus mutans;Lactobacillus casei*	[139]
*Citrus sinensis*	**Antibacterial**: *Escherichia coli*	[140]
*Citrus reticulate*	**Antibacterial**: *Escherichia coli*	[140]
*Citrus limetta*	**Antibacterial**: *Escherichia coli*	[140]
*Citrus medica*	**Antibacterial**: *Pseudomonas aeruginosa*	[141]
*Citrus karna* EO	**Antibacterial**: *Bacillus subtilis*; *Pseudomonas aeruginosa*	[142]
*Annona squamosa*	**Antioxidant**: E showed potent effects	[143]
*Annona reticulate*	**Antioxidant**: E showed potent effects	[143]
Apple (*Malus domestica*)	**Antioxidant**: E showed good effects	[134]
Green sugar apple (*Annona squamosa*)	**Antioxidant**: E showed significant effects	[144]
Purple sugar apple (*Annona squamosa*)	**Antioxidant**: E showed significant effects	[144]
Green star apple (*Chrysophyllum cainito*)	**Antioxidant**: E showed significant effects	[144]
*Apricot (Prunus armeniaca*)	**Antioxidant**: E showed good effects	[134]
*Avocado* (*Persea americana*)	**Antioxidant**: E showed significant effects	[134]
*Grapefruit* (*Citrus x paradisi*)	**Antioxidant**: E showed good effects	[134]
Kiwi (*Actinidia deliciosa*)	**Antioxidant**: E showed good effects	[134,145]
Pomegranate (*Punica granatum*)	**Antibacterial**: *Escherichia coli*; *Proteus vulgaris*; *Pseudomonas aeruginosa*; *Klebsiella pneumonia*; *Staphylococcus saprophyticus*; *Enterococcus faecalis*; *Streptococcus agalactiae*; *Klebsiella pneumoniae*; **Antioxidant**: E showed good effects	[146,147]
Banana (*Musa* sp.)	**Antibacterial**: *Escherichia coli*; *Proteus vulgaris*; *Pseudomonas aeruginosa*; *Klebsiella pneumonia*; *Staphylococcus saprophyticus*; *Enterococcus faecalis*; *Streptococcus agalactiae;***Antioxidant**: E showed significant effects	[146,147]
*lemon* (*Citrus limon*)	**Antibacterial**: *Escherichia coli*; *Proteus vulgaris*; *Pseudomonas aeruginosa*; *Klebsiella pneumonia*; *Staphylococcus saprophyticus*; *Enterococcus faecalis*; *Streptococcus agalactiae*	[146]
*Solanum melongena*	**Antioxidant**: E showed significant effects	[148]
*Putranjiva roxburghii*	**Antibacterial**: *Bacillus subtelis*; *Enterobacter xiangfangensis*; **Antioxidant**: E showed significant effects	[149]
*Pouteria caimito*	**Antibacterial**: *Staphylococcus epidermidis*; *Escherichia coli*	[150]
Saskatoon berry (*Amelanchier alnifolia*)	**Antioxidant**: E showed significant effects	[151]
Rambutan (*Nephelium lappaceum*)	**Antibacterial**: *Salmonella enteritidis; Vibrio parahaemolyticus*; **Antioxidant**: E showed significant effects	[152,153]
*Ficus carica*	**Antibacterial**: *Micrococcus luteus; Proteus vulgaris*; **Antioxidant**: E showed significant effects	[154]
Jaboticaba (*Plinia peruviana*)	**Antioxidant**: E showed significant effects	[30]
Peach (*Prunus persica*)	**Antioxidant**: E showed significant effects	[155]
*Garcia mangostana*	**Antioxidant**: E showed significant effects	[156]
*Persea americana*	**Antioxidant**: E showed significant effects	[156]
*Mangifera odorata*	**Antioxidant**: E showed significant effects	[156]
*Dimocarpus longan*	**Antioxidant**: E showed significant effects	[156]
*Solanum betaceum*	**Antioxidant**: E showed significant effects	[156]
*Annona squamosa*	**Antioxidant**: E showed significant effects	[156]
*Archidendron pauciflorum*	**Antioxidant**: E showed significant effects	[156]
*Parkia speciosa*	**Antioxidant**: E showed significant effects	[156]
*Caryocar brasiliense*	**Antioxidant**: E showed significant effects	[157]
*Leucaena leucocephala*	**Antioxidant**: E showed significant effects	[158]
Loquat (*Eriobotrya japonica*)	**Antioxidant**: E showed significant effects	[159]
Mangosteen (*Garcinia mangostana*)	**Antioxidant**: E showed significant effects	[160]
Rambutan (*Nephelium lappaceum*)	**Antioxidant**: E showed significant effects	[161]
Tea (*Camellia sinensis*)	**Antioxidant**: E showed significant effects	[162]
*Syzygium cumini*	**Antibacterial**: *Staphylococcus aureus; Enterococcus faecalis; Escherichia coli; Pseudomonas aeruginosa; Proteus vulgaris; Serratia marcescens; Bacillus subtilis; Bacillus cereus; Salmonella typhimurium; Enterobacter aerogenes*; **Antifungal**: *Candida albicans; Aspergillus niger*	[163]
Coconut (*Cocos nucifera*)	**Antioxidant**: E showed significant effects	[147]

E: Extract(s); EO: Essential oils.

## 18. Fruit-Peel-Based Edible Coatings/Film and Probiotics

Edible coatings are applied as thin layers on the food surface and results in longer food shelf life, retention of food characteristics, properties and functionality at low cost. Shin et al. [165] developed an apple-peel-based edible coating which was used to keep beef patties fresh. This coating was screened for antioxidant effects towards lipid oxidation along with antimicrobial effects towards yeasts, molds and mesophilic aerobic bacteria. Results demonstrated that this coating treatment inhibited lipid oxidation and effectively suppressed the growth of tested microbial entities on raw beef patties. In another study, Al-Anbar et al. [166] established an orange-peel-based edible coating and this protocol was found to enhance the shelf life of cupcakes. It was found that this coating has the ability to enhance storage age, reduce microbial growth and prevent growth of any yeast or mold during storage time. Moghadam et al. [167] established edible films which were derived from mung bean protein supplemented with pomegranate peel. Of note, this film demonstrated significant antioxidant and antibacterial effects and can be used for the packaging of food products. The combined effects of orange peel (*Citrus sinensis*) essential oil (OPEO) with chitosan film increased the shelf life of fresh shrimps (*Parapenaeus longirostris*) to 15 days [168]. 

In another study, the lemon peel essential oil possessed potent antimicrobial effects against *Escherichia coli* and *Bacillus* sp. and the addition of this essential oil with an edible coating (sodium alginate and cassava starch) significantly decreases the degradation of fresh strawberry and tofu [169]. In addition, nanoemulsion-based edible coatings comprising OPEO can increase the shelf life [170] of orange slices while a combination of gelatin coating and (OPEO) extended shrimp quality by about 6 days [171]. Probiotic yogurt which was prepared with pineapple peel powder enhances antibacterial activity against *Escherichia coli*, as well as anticancer and antioxidant effects [172]. It was discovered that the addition of banana, apple and passion fruit peel powder in probiotic yogurt enhances the growth of *Lactobacillus casei*, *L. acidophilus* and *L. paracasei* [173]. In addition, mango peels exhibit a positive effect in milk supplementation [174] and orange, passion fruit and pineapple peel enhance the firmness and consumer acceptability in yogurt [175]. 

## 19. Conclusions and Future Perspective

Fruit peels, which form a significant portion as a food processing by-product, have not yet been employed as a useful resource for many health supportive products. It is abundantly clear from this review that the potential possibilities to utilize these bioactive components in the food chain are available to the pharmaceutical industry. One of the possible factors for such an unused resource is an erroneous general misconception that fruit peels are unhealthy and considered an undesired waste. The current review has undoubtedly established that fruit peels are indeed a very rich resource of biologically active natural compounds and will have significant benefits for human and animal health. Investing in the fruit peel processing industry, food processors may expand their usability and flexibility to include a fruit-peel-based novel products business venture to establish a profitable existing enterprise. Producing healthy foods with natural ingredients or with fruit peels could bring so many new advantages via the reduction or elimination of food preservatives, artificial additives and replacing them with cheap natural ingredients. In addition, fruit and vegetable peels are being widely employed as food additives and in the modern era of health-conscious young people, such a venture will add to their repertoire of food buying possibilities. 

## Abbreviation

iNOS	Inducible nitric oxide synthase
COX-2	Cyclooxygenase-2
qPCR	Quantitative polymerase chain reaction
HCV 2	Herpes simplex virus 2
VSMC	Vascular smooth muscle cells
PDGF	Platelet-derived growth factor
NOX	(NADPH Oxidase)
NF-κB	Nuclear factor kappa B
IL-1β	Interleukin 1 beta
TNF-α	Tumor necrosis factor-alpha
PGE2	Prostaglandin E2
IL-6	Interleukin 6
